# Do Socio-Demographics Play a Role in the Prevalence of Red Flags and Pursuant Colonoscopies in Patients With Irritable Bowel Syndrome?

**DOI:** 10.7759/cureus.25137

**Published:** 2022-05-19

**Authors:** Anmol Mittal, Shivani Gupta, Faiz Afridi, Anthony Dimitrey, Sushil Ahlawat

**Affiliations:** 1 Department of Internal Medicine, Rutgers University New Jersey Medical School, Newark, USA; 2 Department of Gastroenterology and Hepatology, Rutgers University New Jersey Medical School, Newark, USA

**Keywords:** colonoscopies, socio-demographic disparity, insurance status, red flags, irritable bowel syndrome

## Abstract

Background

Irritable bowel syndrome (IBS) is a “brain-gut disorder” that lacks laboratory, radiologic, or physical exam findings. Colonoscopies are not routinely performed unless “red flag” symptoms, such as bleeding or abnormal weight loss, are present. Socio-demographics have been implicated as sources of potential disparities in appropriate care.

Aims

We hypothesize that the incidence of red flag symptoms and pursuant colonoscopies differ by socio-demographic status in patients with IBS.

Methods

Patients diagnosed with IBS were extracted from the National Inpatient Sample 2001-2013 using the International Classification of Diseases, Ninth Revision (ICD-9) codes. Gastrointestinal bleed, blood in stool, weight loss, and anemia were pooled into red flag symptoms. Colonoscopies during the admission were identified using ICD-9 procedural codes. Chi-square analysis and binomial logistic regression were used to evaluate potential disparities with α<0.01.

Results

Patients with Medicaid or Medicare or those without insurance had higher odds of presenting with red flag symptoms compared to those with private insurance. Medicaid patients and uninsured patients had higher odds of undergoing colonoscopies. All patients that were not Caucasian had higher odds of presenting with red flags and subsequently undergoing colonoscopies. Older patients had higher odds of presenting with concerning red flag symptoms but lower odds of undergoing colonoscopies.

Conclusions

The incidence of red flag symptoms and performance of colonoscopies differed by socio-demographics in patients with IBS. Patients with non-private or those without insurance were more likely to have red flags and undergo a colonoscopy. Age and race also increased rates of red flag symptoms while having a mixed effect on pursuant colonoscopies. This may represent discrepancies in healthcare utilization in a vulnerable population.

## Introduction

Irritable bowel syndrome (IBS) is a functional gastrointestinal (GI) disorder consisting of abdominal pain associated with diarrhea, constipation, or both. Nearly $30 billion are spent every year on national direct and indirect costs to treat IBS [1-2]. Irritable bowel syndrome is currently diagnosed based on clinical symptoms only using various criteria. The 2006 Rome III criteria define IBS as three months of recurrent abdominal pain that occurs at least one day per week and is associated with at least two of the following: defecation, change in frequency of stool, and change in form of stool. Additionally, symptom onset should be six months prior to diagnosis [3]. In the United States, about 10% of the population fulfills the Rome III criteria for IBS, making it a relatively common condition [4]. More extensive testing is advocated for patients exhibiting alarm signs, which include unexplained weight loss, rectal bleeding, family history of bowel cancer, fever, and anemia [1].

The American College of Gastroenterology recommends performing colonoscopies in patients that present with IBS and alarm features to exclude organic bowel diseases. They also recommend performing colonoscopies in patients over 50 years of age to screen for colorectal cancer. Outside of these two scenarios, colonoscopies are not recommended due to the low prevalence of Crohn's disease, ulcerative colitis, and colonic neoplasia [5].

IBS has been demonstrated to be more prevalent in females, however, previously performed meta-analyses looking at socio-demographics, in their limited scope, did not find any correlation [6-7]. Other studies have demonstrated the importance of race as a consideration for IBS and the implications of racial disparities, however, they did not address the disparities associated with the concerning symptoms [8]. Though there are data to suggest that socio-demographics such as race can affect the type of care received for IBS, including a lack of gastroenterology consults or treatment by specialists, there are no studies that have studied the presence of concerning red flag symptoms in this affected patient population [9]. Certain comorbidities associated with IBS, including anxiety and depression, have also been demonstrated to have an association with socioeconomic status [10]. Insurance status has also been identified as an enabling factor that impacts health care utilization, typically delaying presentation and often resulting in uninsured patients demonstrating higher rates of complication and mortality [11]. Specifically, IBS patients with health insurance have had a higher probability of utilizing medical healthcare services than those patients without insurance [4,12].

It has not yet been identified in the literature if socio-demographic status affects the incidence of alarming features in IBS or the rates of pursuant colonoscopies. This paper aims to explore if the presentation of red flag symptoms, which leads to colonoscopies, is related to socio-demographics for patients with IBS.

This article was previously presented as a meeting abstract at the 2020 American College of Gastroenterology Annual Meeting on October 23-28, 2020.

## Materials and methods

Data source

The National Inpatient Sample (NIS) database was utilized to perform a retrospective cohort analysis of the inpatient admissions from 2001 to 2013. The NIS database is part of the healthcare cost and utilization project, which has been used in prior research as an effective tool to measure all-payer inpatient data. Data are collected from over seven million hospital stays each year, which represents 20% of the admission from 47 states, including the District of Columbia, covering 97% of the U.S. population. Included in the data set are multiple elements, including admission diagnoses, procedures performed, the length of stay, mortality events, and payor source. Institutional Review Board (IRB) approval was not obtained as the NIS database is de-identified.

Study population 

The study population was set to include patients older than 18 years of age, who were admitted to the hospital, and had a primary diagnosis (DX1 - DX3) of IBS using the coding in accordance with the International Classification of Diseases, 9th revision (ICD-9) in the NIS database from 2001 to 2013 (Table 1). All adults with IBS who underwent an inpatient colonoscopy were identified using ICD-9 procedure codes (Table 1).

**Table 1:** International Classification of Diseases, Ninth Revision (ICD-9) diagnostic and procedure codes

ICD-9 diagnostic and procedure codes	
ICD-9 diagnostic code	Diagnosis
564.1	Irritable bowel syndrome
578.9	Hemorrhage of the gastrointestinal tract
578.1	Blood in stool
783.2	Abnormal loss of weight and underweight
783.21	Loss of weight
285.9	Anemia, unspecified
280	Iron deficiency anemia
280.0	Iron deficiency anemia secondary to blood loss (chronic)
280.9	Iron deficiency anemia, unspecified
ICD 9 procedure code	Procedure description
45.23	Colonoscopy
45.24	Flexible sigmoidoscopy
45.25	Closed endoscopic biopsy of the large intestine
45.42	Endoscopic polypectomy of the large intestine
48.23	Rigid proctosigmoidoscopy
48.24	Closed endoscopic biopsy of the rectum
48.36	Endoscopic polypectomy of the rectum

Study variables/outcomes

The goal of this study was to understand the influence of demographic variables such as insurance status on the evaluation of alarming red flag symptoms in patients with irritable bowel syndrome and the evaluation using a colonoscopy. Patients with symptoms of hemorrhage in the gastrointestinal tract, blood in stool, loss of weight, abnormal loss of weight, and anemia unspecified were attributed to be key red flags that were grouped into a single category as red flags identified by ICD-9 codes (Table 1). Patient demographics, including age, sex, income, and insurance status, were identified. Finally, hospital utilization factors, such as length of stay and hospital costs, were also analyzed.

Hospital charges

Hospital charges are reported by year and therefore were adjusted for inflation. The mean charge was adjusted to the December 31, 2013, equivalent rate using the U.S. Bureau of Labor Statistics Inflation Calculator [13].

Statistical analysis

Statistical analysis was performed using SPSS (software version 28), IBM Corp., Armonk, NY. Chi-square analysis was performed on the categorical data to determine variables to be included in the analysis. A binary logistic regression analysis was used to assess the socio-demographic variables for patients with IBS. Results from this analysis were then used to perform a propensity-matched logistical regression analysis to understand the relationship of socio-demographics to red flag symptoms. This same method was used to evaluate the association between colonoscopies performed and the socio-demographic variables. An analysis of variance (ANOVA) test with a test of linearity was done to perform trend analysis for the mean length of stay and the hospital costs by year for patients presenting with IBS. All statistical tests were performed with a significance level of α <0.01. 

## Results

Patient characteristics and demographics

**Figure 1:**
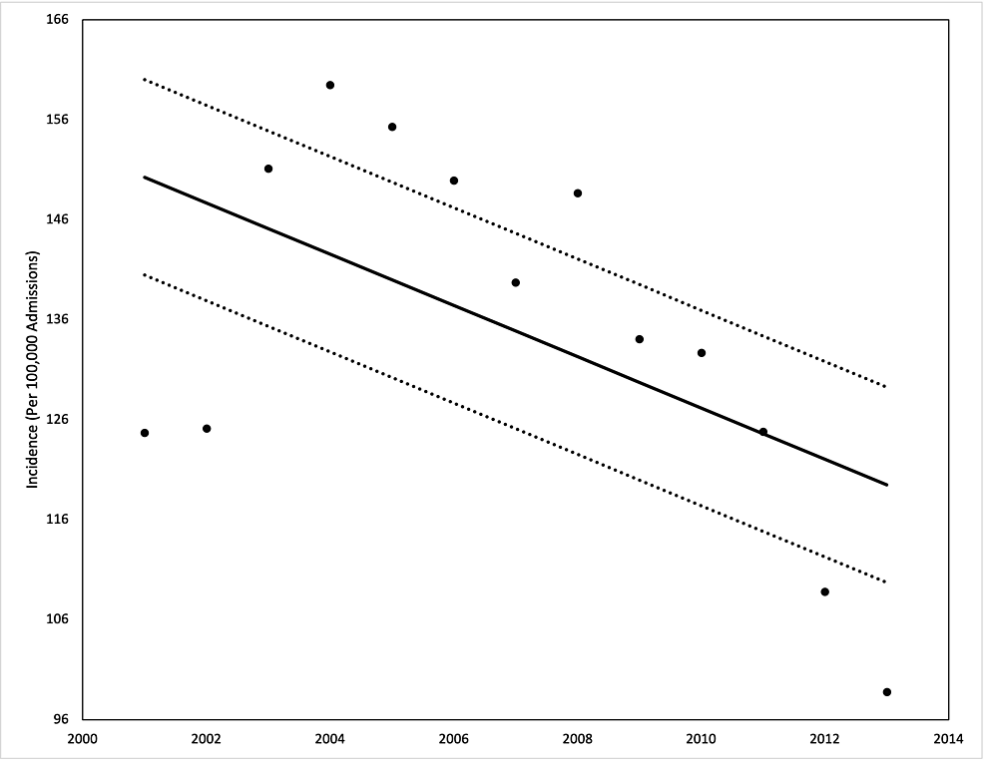
Incidence of irritable bowel syndrome in our patient population from 2001 to 2013 Analysis of variance (ANOVA) with linear trend analysis (solid line) and 95% confidence interval (dashed line) visualized R^2 ^= 0.297, p = 0.09

There were 655,807 patients with a diagnosis of IBS identified in the NIS database, which is equivalent to a national estimate of approximately 3,300,000 IBS patients in a 13-year period or approximately 254,000 patients per year. Charting the incidence per 100,000 admissions demonstrates an overall non-significant but negative trend with an R^2^ value of 0.30 (Figure 1). The demographics of the patients that presented with red flag symptoms and those that did not are presented in Table 2. Using chi-squared analysis, there were significant differences between the two populations between the different age groups, the different race groups, median incomes, and the patients who received colonoscopies. There was no difference between the sex of the patients. Of the patients with IBS, 65,220 (9.9%) presented with red flag symptoms during the stay, and there were 108,484 (16.5%) who had a colonoscopy during their admission.

**Table 2:** Demographic data for patients admitted from 2001 to 2013 with irritable bowel syndrome *Significance level p<0.01

Variable	No symptoms	Red flag symptoms	P – value
Age			<.001^*^
19 to 30	12.3%	11.8%	
31 to 50	40.7%	36.7%	
51 to 60	17.9%	15.7%	
61 to 79	21.8%	24.0%	
≥ 80	7.3%	11.7%	
Race			<.001^*^
Caucasian	83.9%	75.9%	
African American	6.7%	12.4%	
Hispanic	6.3%	7.6%	
Asian, Pacific Islander, Native American	3.1%	4.0%	
Gender			<.001^*^
Males	19.4%	17.3%	
Females	80.6%	82.7%	
Insurance status			<.001^*^
Private insurance	51.6%	43.5%	
Medicaid	10.6%	11.7%	
Medicare	28.9%	35.0%	
No insurance	5.6%	6.4%	
Other insurance status	3.4%	3.4%	
Median Income Quartiles			<.001^*^
0-25th percentile	19.0%	21.6%	
26-50th percentile	24.5%	24.1%	
51-75th percentile	26.5%	26.5%	
76-100th percentile	30.0%	27.9%	
Colonoscopy			<.001^*^
No colonoscopy	85.1%	68.5%	
Had colonoscopy	14.9%	31.5%	

Healthcare trends for admitted IBS patients

**Figure 2:**
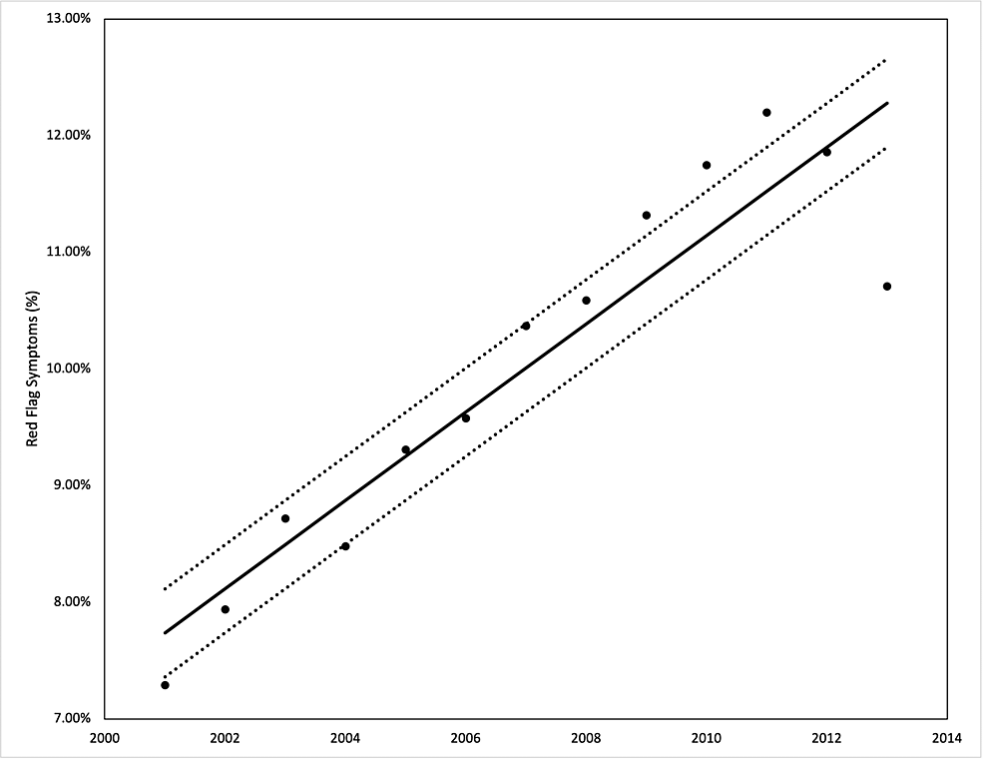
Incidence of red flag symptoms in our patient population from 2001 to 2013 Analysis of variance (ANOVA) with linear trend analysis (solid line) and 95% confidence interval (dashed line) visualized R^2^ = 0.861, p = 0.001

**Figure 3:**
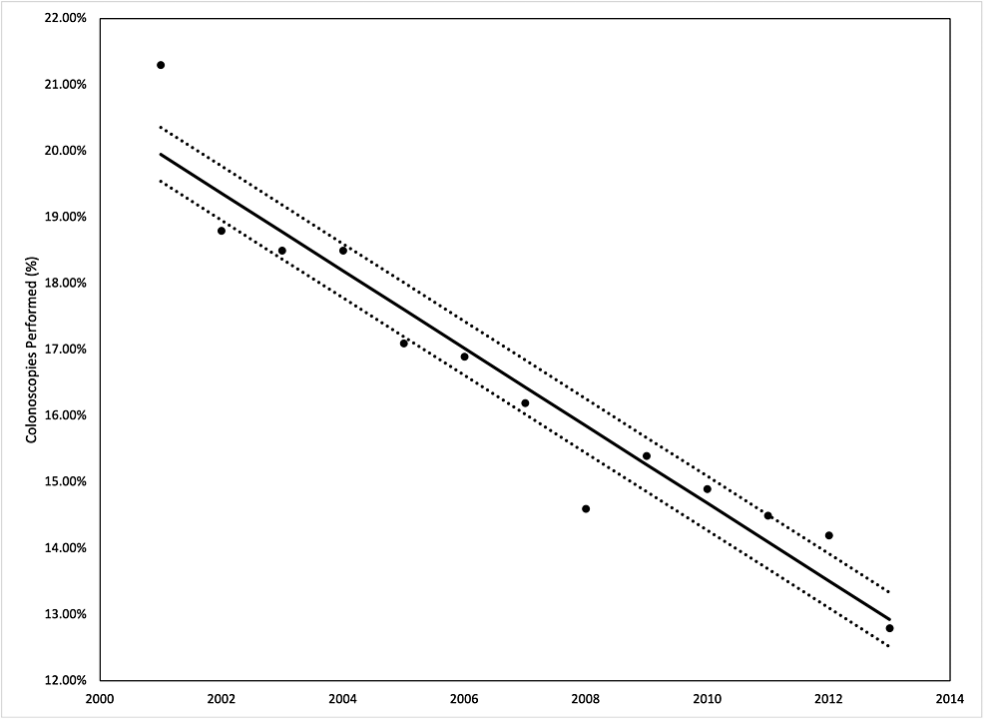
Percentage of pursuant colonoscopies performed in our patient population from 2001 to 2013 Analysis of variance (ANOVA) with linear trend analysis (solid line) and 95% confidence interval (dashed line) visualized R^2^ = 0.927, p = 0.001

**Figure 4:**
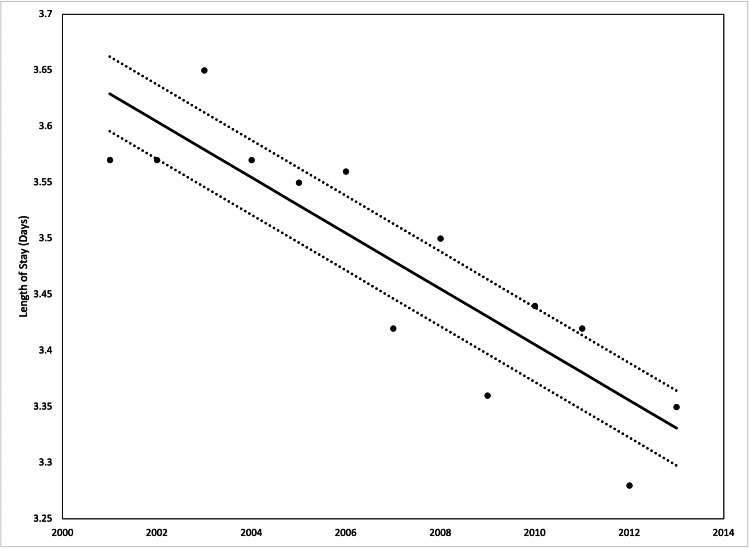
Average length of stay by year in our patient population from 2001 to 2013 Analysis of variance (ANOVA) with linear trend analysis (solid line) and 95% confidence interval (dashed line) visualized R^2^ = 0.774, p = 0.001

**Figure 5:**
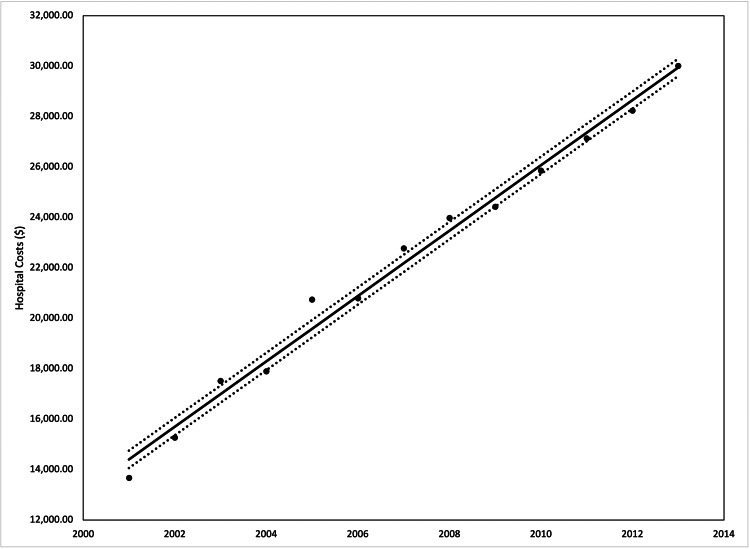
Average hospital costs (corrected for inflation) by year in our patient population from 2001 to 2013 Analysis of variance (ANOVA) with linear trend analysis (solid line) and 95% confidence interval (dashed line) visualized R^2^ = 0.989, p = 0.001

The incidence of red flags from 2001 to 2013 increased from 7.3% to 10%, however, the performance of colonoscopies in the patients decreased from 21.3% to 16.5%. The average length of stay from 2001 to 2013 decreased from 3.5 days to 3.3 days, but the average hospital costs increased from $10,278 in 2001 to $19,076 in 2013. Both were statistically significant with a p-value <0.001 from ANOVA (Figures 2-5).

Prediction of red flag symptoms

Using patients aged 19 to 29 as the reference, patients aged 30 to 50 and 51 to 60 were not found statistically significantly to have any differences in the occurrence of red flag symptoms, though, for the younger cohort, red flag symptoms occurred less often non-significantly. Patients aged 61 to 79 and great than 80 years old were found to have an increased odds of presenting with red flag symptoms. Those aged 61 to 79 were 1.14 times more likely to have red flag symptoms, and those aged greater than 80 were 1.63 times more likely to have red flag symptoms. Patients of any race as compared to Caucasians were more likely to have red flag symptoms. African Americans were 2.1 times more likely, Hispanic patients were 1.4 times more likely, and Asian, Pacific Islander, or Native American descents were 1.2 times more likely to have red flag symptoms. Females in the study sample were 1.2 times more likely to have red flag symptoms. With respect to median incomes, it was found that compared to the 0-25th percentile, the other quartiles had no statistically significant decreased odds of red flag symptoms. Patients with all insurances other than private insurance were more likely to have red flag symptoms. Patients with Medicaid and Medicare were 1.2 times more likely to have red flag symptoms. Those that were uninsured were 1.3 times more likely to have red flag symptoms (Table 3).

**Table 3:** Socio-demographic predictors of red flag symptoms in admitted IBS patients IBS: irritable bowel syndrome *Significance level p<0.01

Variable	P-value	Odds ratio (95% CI)
Age		
19 to 30	Reference	
31 to 50	0.417	0.97 (0.91-1.04)
51 to 60	0.280	0.96 (0.88-1.04)
61 to 79	0.002^*^	1.14 (1.05-1.24)
≥ 80	<0.001^*^	1.63 (1.48-1.81)
Race		
Caucasian	Reference	
African American	<0.001^*^	2.08 (1.94-2.22)
Hispanic	<0.001^*^	1.36 (1.26-1.47)
Asian, Pacific Islander, Native American	<0.001^*^	1.18 (1.11-1.24)
Gender		
Males	Reference	
Females	<0.001^*^	1.18 (1.11-1.24)
Insurance status		
Private insurance	Reference	
Medicaid	<0.001^*^	1.18 (1.10-1.27)
Medicare	<0.001^*^	1.19 (1.12-1.26)
No insurance	<0.001^*^	1.30 (1.19-1.42)
Other insurance status	<0.001^*^	1.23 (1.10-1.39)
Median income quartiles		
0-25th percentile	Reference	
26-50th percentile	0.030	0.94 (0.88-0.99)
51-75th percentile	0.279	0.97 (0.91-1.03)
76-100th percentile	0.009^*^	0.92 (0.87-0.98)

Prediction of pursuant colonoscopies 

Patients aged 30 to 50, 51 to 60, 61 to 79, and those greater than 80 were all found to have statistically significantly decreased odds of undergoing a colonoscopy compared to ages 19 to 29. Patients aged 30 to 50 had an odds ratio of 0.9, 51 to 60 had an odds ratio of 0.8, 61 to 79 had an odds ratio of 0.7, and ages greater than 80 had an odds ratio of 0.7. Compared to patients of Caucasian descent, African Americans were 1.2 times more likely, Hispanics were 1.3 times more likely, and Asian, Pacific Islander, and Native Americans were 1.1 times more likely to have a colonoscopy performed. Patients that were females were less likely to get a colonoscopy at an odds ratio of 0.8. Patients, as compared to the 0-25th percentile, of all median income quartiles did not have any significant increase in odds of undergoing a colonoscopy. Compared to patients with private insurance, those with Medicare did not have a statistically significant difference in odds of getting a colonoscopy but those with Medicaid and those without insurance were 1.1 and 1.6 times more likely, respectively (Table 4).

**Table 4:** Socio-demographic predictors of inpatient colonoscopies in admitted IBS patients with red flag symptoms IBS: irritable bowel syndrome *Significance level p<0.01

Variable	P-value	Odds ratio (95% CI)
Age		
19 to 30	Reference	
31 to 50	<0.001^*^	0.89 (0.84-0.94)
51 to 60	<0.001^*^	0.77 (0.72-0.81)
61 to 79	<0.001^*^	0.74 (0.69-0.79)
≥ 80	<0.001^*^	0.69 (0.63-0.76)
Race		
Caucasian	Reference	
African American	<0.001^*^	1.24 (1.17-1.32)
Hispanic	<0.001^*^	1.33 (1.24-1.41)
Asian, Pacific Islander, Native American	0.008^*^	1.13 (1.03-1.24)
Gender		
Males	Reference	
Females	<0.001^*^	0.84 (0.80-0.87)
Insurance status		
Private insurance	Reference	
Medicaid	<0.001^*^	1.13 (1.07-1.19)
Medicare	0.219	0.97 (0.92-1.02)
No insurance	<0.001^*^	1.53 (1.43-1.64)
Other insurance status	0.237	0.94 (0.86-1.04)
Median income quartiles		
0-25th percentile	Reference	
26-50th percentile	0.135	1.04 (0.99-1.09)
51-75th percentile	0.677	0.99 (0.94-1.04)
76-100th percentile	0.301	1.03 (0.98-1.08)
Red flag symptoms		
Not present	Reference	
Present	<0.001^*^	2.49 (2.38-2.61)

## Discussion

Irritable bowel syndrome, though benign in pathology, is an enigmatic disease connected across many disciplines, including gastroenterology, psychiatry, and medicine. Patients with irritable bowel syndrome experience severe distress and disability and often are not treated appropriately due to the nonspecific constellation of symptoms, which can lead to a high healthcare utilization burden [14].

In this study, we found that the incidence of red flag symptoms and performance of colonoscopies differs by socio-demographics in patients admitted with IBS. Currently, there are many conflicting studies understanding the effect of socio-demographics; however, from this large inpatient sample, it is evident that those without insurance present more often with red flag symptoms and as a result are more likely to get colonoscopies. Those of any race other than Caucasian also have a predilection for presenting with red flag symptoms and therefore undergoing colonoscopies. The implications of this emphasize the ongoing evaluation of the social disparities present in our healthcare system.

Age was also a contributor to the disparities in patients admitted for IBS as compared to the second decade of life. Those individuals 30 to 60 years old did not show any differences, but those older than sixty were more like to demonstrate these red flag symptoms. It is reasonable to consider that older patients may be more likely to present with anemia or lower bleeding due to other comorbidities, such as polyps or diverticular disease; however, in our study, it was observed that all ages were less likely to undergo colonoscopy compared to the 19-30 age population [15]. This is unexpected as typically older patients are more likely to undergo inpatient colonoscopies compared to younger patients [16]. This disparity may be a result of a higher level of concern for these alarming symptoms being atypical or more likely concerning for inflammatory bowel disease in the younger population as compared to the older. Younger patients are more likely to present with these red flag symptoms and more likely to undergo colonoscopies. Given that these younger patients are more likely to be uninsured, as they don't qualify for government assistance for health insurance, the lack of insurance may contribute to such disparity in red flag symptoms and pursuant colonoscopies [17].

There is currently minimal research comparing socio-demographic status with red flag symptoms or colonoscopies in patients with IBS. To our knowledge, this is the largest and most comprehensive analysis of factors associated with colonoscopies in patients with IBS and as they relate to demographic factors. Patients with non-private insurance and those who are uninsured are more likely to experience red flag symptoms compared to those with private insurance, and those with Medicaid and no insurance are also more likely to undergo a colonoscopy.

Endoscopic procedures like colonoscopy constitute 50-75% of the entire cost of the workup for IBS, making it the most expensive portion [18]. Despite the current colorectal cancer recommendation that normal screening colonoscopies should be held every 10 years, nearly 50% of Medicare patients had a repeat colonoscopy in less than seven years without any clear indication for the early examination [19-20]. This reflects a potential overuse of screening colonoscopies, creating a financial burden and increasing patient mortality [19]. Based on our results, a healthcare utilization discrepancy may also be seen in patients with IBS who are either uninsured or receiving Medicaid, as we found that they are more likely to undergo a colonoscopy. As observed in other studies, we also observed a negative trend in the number of patients diagnosed with IBS undergoing colonoscopies inpatient as well as the average length of stay, which may demonstrate increased proficiency in understanding IBS and the risk of observation in this patient population, however, our trends demonstrated a continued increase in hospital costs [21]. This trend demonstrates the importance of preventing hospitalizations and further exploring treatment options.

IBS typically has three modalities of treatment, including nutritional therapy, drug therapy, or psychotherapy. Although there has been a lack of studies to demonstrate the effects of vitamins and digestive enzymes, there have been many subjective reports of improvement. The use of prebiotics and probiotics are now being subjected to further studies, as they may have a significant role in altering the gut flora. The balancing act, however, is the cost of these medications as the exploitation of “natural remedies” has driven the demand. Shown to decrease the overall healthcare costs by decreasing additional medications, requiring consulting different services, and decreasing hospital length of stay, treatment with these medications may benefit patients who may not be able to afford their admission to the hospital [22-23]. Drug therapy also reflects this increased focus on the alteration of gut microbiota. Drug therapy initially involved antispasmodic drugs, low-dose antidepressants, and laxatives or motility accelerants. However, there is now a focus on agents that alter the microbiome, including medications such as rifaximin. Finally, the last treatment arm of this disease includes the use of psychotherapy to address the “brain” aspect of the “brain-gut syndrome.” Patients are known to have somatic symptoms that are an extension of the patient’s negative feelings. With psychotherapy, patients can reduce psychological stress, which improves their brain-gut signaling. However, the economic burden of all these medications and treatments makes them less viable, especially to those with a low socioeconomic status. The inability to access these medications continues the positive effect of delaying presentation and presenting with the various red flag symptoms [23].

## Conclusions

Irritable bowel syndrome, a disease of the "brain-gut axis," is a very common disorder that has yet to have a definitive treatment. Though experienced by many, studies demonstrate the unequal distribution of IBS, as it affects certain demographics disproportionately. The goal of this study was to understand different factors that sway the decision to perform a colonoscopy on a patient with IBS. As the American College of Gastroenterology (ACG) guidelines recommend, a colonoscopy is typically only performed when there is the presence of red flag symptoms such as weight loss, luminal blood loss, or anemia. Through the analysis of this large inpatient database, it was found that uninsured patients were more likely to present red flag symptoms and undergo colonoscopies. All non-Caucasian races were also at high risk for presenting with red flag symptoms and undergoing colonoscopies. Finally, we found that a patient's older age predisposed them to a high risk of red flag symptoms; they underwent colonoscopies at a lower rate as compared to their younger counterparts. The importance of understanding the role socio-demographic factors play in patients with IBS is underrated, as we demonstrate the higher hospital utilization costs attributed to performing more colonoscopies due to increased occurrences of red flag symptoms. Further research must be done to understand how to mitigate the socio-economic disparities present to improve patient outcomes and hospitalization costs.
